# Linking field-based mobility tests to match-derived performance in wheelchair rugby: understanding test-match agreement across impairment types

**DOI:** 10.3389/fspor.2026.1878412

**Published:** 2026-07-01

**Authors:** Rienk M. A. van der Slikke, Viola C. Altmann, Mariska M. H. P. Janssen, Monique A. M. Berger

**Affiliations:** 1 Centre of Expertise Health Innovation, Research Group Assistive Technology for Mobility and Sports, The Hague University of Applied Sciences, The Hague, Netherlands; 2 Department of Biomechanical Engineering, Delft University of Technology, Delft, Netherlands; 3 Peter Harrison Centre for Disability Sport, School of Sport, Health and Exercise Sciences, Loughborough University, Loughborough, United Kingdom; 4 Klimmendaal Rehabilitation Specialists, Arnhem, Netherlands

**Keywords:** classification, paralympics, sports performance, trunk function, wheelchair athletes

## Abstract

Field-based performance and skill tests are widely used in wheelchair rugby (WR) to assess physical capacity, yet their relation to in-game mobility and potential differences between athletes with and without coordination impairment (CI) remain unclear. This study examined (1) ecological validity and test-match correspondence of a comprehensive WR field-test battery, reflecting maximal capacity, against match-derived mobility performance, (2) impairment-based differences in test-match agreement, and (3) trunk-movement differences between CI and Non-CI athletes. Fifty-two international WR athletes (*N*on-CI: *n* = 27; CI: *n* = 25) completed a standardized battery (sprint, turning, stop-go, complex skills) with wheel- and trunk-mounted inertial sensors. Match metrics (e.g., average/maximal speed, rotational speed) were derived from full-match IMU data. Test-match correspondence was assessed using Pearson correlations and Lin's concordance; agreement with Bland–Altman analysis; group differences with Welch's *t*-tests (FDR/Holm corrected). Trunk angle and trunk-relative accelerations were analyzed separately, with classification included in supplementary analyses. Maximal forward speed showed strong association (20 m sprint: *r* = 0.878, small bias), and rotational capacity was best captured by an isolated 180° turn (*r* = 0.796). Acceleration metrics showed moderate correlations but poor absolute agreement, indicating they reflect maximal capacity rather than match-equivalent output. CI athletes showed smaller test-match discrepancies, whereas Non-CI athletes’ tests tended to underestimate match speed and overestimate acceleration. Trunk-sensor outcomes differed strongly between groups (*g* ≈ 0.9–1.2), indicating substantial impairment-related variation. Classification correlated positively with performance, with similar strength across groups. Linear sprint and isolated turning tests show strong associations with in-game WR performance, while acceleration metrics mainly index maximal capacity. Field tests appear to align more closely with match behavior in CI athletes than in Non-CI athletes. Trunk-sensor measures add value for profiling and may support future classification.

## 1 Introduction

Performance assessment is essential for optimizing training, talent identification, and competition strategies in elite team sports. In wheelchair rugby (WR), field-based tests such as sprints, turns, and agility tasks are commonly used to assess wheelchair mobility capacities that are considered relevant for match play. These capacities reflect different aspects of wheelchair mobility performance, which can be conceptualized as distinct but related performance domains, including maximal speed, rotational capacity, and acceleration-based outputs. However, both the ecological validity and test–match correspondence of field-based tests across these domains may vary and may depend on athlete characteristics and impairment profiles (1, 2).

A key assumption in this context is that the interpretation of field-based wheelchair mobility tests implicitly assumes that the relationship between test performance and in-game behavior is comparable across impairment types. Differences in movement control, trunk involvement and coordination may alter how maximal capacities measured in standardized tests translate to actual match actions. As a result, identical test outcomes could have a different ecological meaning for athletes with and without coordination impairment (CI). Importantly, field-based tests do not separate impairment-related limitations from trainable physical capacities, but capture their combined expression under standardized conditions. Different performance domains may therefore vary in the extent to which their test outcomes are constrained by impairment, influenced by training, or modulated by contextual match demands. As a result, the ecological meaning of identical test outcomes may differ across impairment types.

WR presents additional challenges in this respect due to its dynamic, multidirectional demands and the heterogeneity of impairments within a team. In particular, athletes with CI appear to represent a distinct subgroup, characterized by different movement patterns and performance outcomes compared to athletes with other impairment types (2–4). Historically, athletes with CI have been underrepresented in wheelchair rugby relative to other eligible impairment types, partly reflecting uncertainties regarding eligibility, classification, and performance comparability, as described in the IPC classification code (5). Recent findings indicate that the participation of athletes with CI at elite level is increasing, further emphasizing the need to critically examine how field-based testing, training practices, and classification procedures apply to this specific athlete group (3). Whereas earlier work using part of this dataset focused on activity limitations and classification-related differences between impairment groups (3), the present study specifically examines how field-test outcomes translate to match-derived mobility performance and whether this translation differs between athletes with and without coordination impairment. Such differences have broader implications beyond performance assessment, particularly for athlete classification. Classification systems aim to reflect the impact of impairment on performance, yet their validity partly depends on the extent to which commonly used field tests capture sport-specific performance in a comparable manner across impairment types. Previous work has primarily focused on the general validity of field tests in wheelchair sports but less is known about whether this validity holds equally across impairment groups.

Advances in sensor technology enable detailed measurement of kinematic variables during both standardized testing and competition, offering opportunities to examine these relationships in greater detail (4, 6). The present study therefore investigates the associations between a comprehensive field-test battery and match-derived mobility performance, with a specific focus on differences between athletes with and without CI. Classification level is considered as a contextual variable to evaluate whether observed impairment-related patterns are independent of classification. The findings aim to inform more tailored evaluation protocols that enhance the validity and interpretability of performance assessments across diverse athlete profiles.

## 2 Methods

### 2.1 Procedures

This study included 52 elite wheelchair rugby (WR) athletes, representing all functional classes, with 27 non-CI athletes and 25 CI athletes (Table 1). The Non-CI group represents a heterogeneous set of impairments with differing functional consequences, which should be considered when interpreting between-group comparisons. All participants played WR at an international level and were recruited and measured during one of three international tournaments: the Wheelchair Rugby World Championship 2022, the Musholm Cup 2023, and the Amsterdam Quad Rugby Tournament 2023. Prior to participation, all athletes provided written informed consent, including consent to use their classification data from the classification database. Ethical approval for the study was obtained from the ethics committee of Delft University of Technology (v1895_2022).

**Table 1:** Participant overview.

Group	Sex (F/M)	Class (SD)	Underlying health condition
Total	7/45	2.1 (0.9)	
Non-CI	2/25	2.0 (1.0)	SCI (16), amputee (2), Limb def. (2), CMT (3), VACTERL (1), CSD (1), MD (1), AMC (1)
CI	5/20	2.2 (0.8)	CP (24), HSP (1)

The underlying health conditions as documented in the classification files, with SCI, spinal cord injury; CMT, Charcot-Marie-tooth disease; AMC, arthrogryposis multiplex congenita; MD, muscular dystrophy; CSD, caudal spine defect; HSP, hereditary spastic paraplegia; CP, cerebral palsy.

Field based performance tests were conducted in the athlete's own sports wheelchair, with the same matchlike setup and on the same surface type as the recorded match. The test battery was designed to represent key components of wheelchair rugby performance and included straight line sprinting, stop-start drills, turning maneuvers, and slalom like movement sequences (7). The main elements consisted of a 20 m forward sprint (TP1), a 12 m sprint with a complete stop (TP2), a 12 m sprint with repeated stop-go transitions at 3 and 6 meter (TP3), a combined sprint-turn-slalom task (TP4), and several 180 degree turning tasks performed on the spot (TP5 = turn right; TP6 = turn left; TP7 = turn right with stop a go at 90 degrees; TP8 = like TP7 but to the left). Two more complex skill-based courses (X-test), one with (TP9) and one without ball handling (TP10), were also included.

During all field tests and matches, wheelchair mobility performance was measured using an inertial measurement unit (IMU) mounted on the right wheel (6, 8). During the field tests, an additional IMU was worn on the trunk at approximately C7 height to quantify trunk movement and orientation. This IMU was used to quantify mean trunk angle and forward or backward trunk accelerations relative to the wheelchair. These trunk related measures provide insight into postural control and movement strategy in the field tests. All match data and nearly all field tests were recorded using xIMU3 sensors (Bristol, UK). Due to practical constraints, five field-test recordings were obtained using Movesense HR + sensors (Vantaa, Finland), which have comparable specifications in terms of IMU accuracy. All sensors recorded data at a sample frequency of 100 Hz.

### 2.2 Data analysis

A custom-built Python script (v3.11) was used to process the IMU data into wheelchair mobility performance outcomes. From the wheel-mounted IMU recordings during matches, reference performance indicators were derived, including maximal forward and rotational speed, mean forward and rotational acceleration, and acceleration per push during phases exceeding the average speed threshold of 1.1 m/s in WR (2). These match-derived metrics served as context-dependent reference values for comparison, reflecting in-game performance rather than an independent gold standard, and where used for comparison with corresponding outcomes obtained from the field-based tests.

### 2.3 Outcome measures

Performance variables for both match play and field tests were derived from the IMUs and processed using identical filtering and thresholding procedures. Wheelchair kinematics were obtained from the IMU by converting quaternion data to Euler angles and with a correction for sensor misalignment (9). Angular rates were median-filtered and low pass filtered (5–20 Hz), and forward speed was derived from wheel roll rate with a correction for pivot related turning contributions (10). For match analysis, a movement segment was defined as a sequence of samples with |speed| ≥ 0.1 m/s and rotation segment as |rotational speed| ≥ 10 deg/s (6). From these signals, maximal and mean forward speeds were calculated, along with their “best five” variants based on the highest values across movement segments. Mean forward acceleration was obtained as the average absolute derivative of speed within movement periods. Maximal and mean rotational speeds, and mean rotational acceleration, were computed analogously using rotation in the horizontal plane and its derivative. Acceleration per push was calculated using a standardized push-detection algorithm (10). Push accelerations were derived from changes in filtered speed over each push. During phases where instantaneous speed exceeded the WR mean speed (1.1 m/s), accelerations per push were summarized, and the five highest values were selected as match-reference measures. Match-derived performance metrics were calculated from full competitive match recordings and therefore inherently reflect the constraints imposed by tactical play, defensive pressure, and physical contact with opponents. The reliability of the IMU-derived wheelchair mobility outcomes has been established in previous studies using similar sensor configurations and analytical approaches, demonstrating good to excellent consistency for speed- and acceleration-based metrics (6, 8, 10). Although reliability was not re-assessed within the present dataset, these prior findings support the robustness of the performance measures derived.

Field test variables were computed using the same signal processing pipeline but within predefined task windows for each test part. For every field test, maximal and mean forward speed, mean forward acceleration, maximal and mean rotational speed, and mean rotational acceleration were calculated in direct analogy to the match definitions to allow one to one comparison. During field tests, an additional trunk mounted IMU provided mean trunk angle and trunk relative accelerations (forward and backward). Average trunk angle was defined as the time-averaged sagittal trunk inclination (in degrees) relative to the wheelchair frame, computed over the full duration of each test part (TP), with 90° a corresponding to a vertical trunk, and 0° fully flexed. The forward and backward accelerations were computed by rotating the trunk accelerations into a global frame and subtracting wheelchair frame accelerations. These trunk-related measures were included to characterize impairment-related movement strategies and to support interpretation of group-level differences in test-match agreement, rather than to model individual test-match discrepancies.

For analytical purposes, wheelchair mobility performance was organized into five performance domains, each representing a distinct functional aspect of wheelchair movement relevant to match play: (1) maximal forward speed, (2) maximal rotational speed, (3) mean forward acceleration, (4) mean rotational acceleration, and (5) acceleration per push. To obtain a single field-test estimate per athlete and performance domain, a TestMax value was calculated. TestMax was defined as the highest value achieved across all relevant field-test parts (TPs) within a given domain (e.g., the maximal speed attained in any sprint- or agility-related task). This approach provides a standardized estimate of maximal expressed capacity during field testing and enables direct comparison with match-derived reference values. The use of TestMax aligns with common practice in sport performance testing, where maximal expressed performance is considered indicative of an athlete's physical capacity. However, this approach emphasizes peak values and may be sensitive to measurement noise or extreme observations, while not capturing within-athlete variability. As such, TestMax should be interpreted as an estimate of maximal capacity rather than typical performance.

### 2.4 Statistical analysis

Assumptions of linearity were evaluated visually using scatterplots, and no dominant outliers were observed. First, test-match correspondence between field-test outcomes and their corresponding match-derived performance metrics was examined using Pearson's correlation coefficient. Pearson's r was chosen because the relationship of interest concerns the extent to which athletes who perform well in a field test also perform well in match play, irrespective of absolute scale differences. To complement r, Lin's Concordance Correlation Coefficient (CCC) was computed, as CCC accounts for both correlation and systematic mean differences and therefore summarizes agreement in both scale and location, offering a stricter criterion than Pearson's r when evaluating measurement equivalence. Reporting both r and CCC allows rank-order association and absolute agreement to be interpreted separately. Second, to evaluate absolute agreement between matched test and match values, Bland–Altman analysis was applied (11). Bland–Altman plots quantify systematic bias (Test - Match) and 95% limits of agreement (LoA), providing insight into whether field tests can reproduce match-like magnitudes at the individual level. Bland–Altman plots were visually inspected for proportional bias and heteroscedasticity, which were interpreted descriptively where present. This combination of relative (r, CCC) and absolute (bias, LoA) metrics provides a comprehensive assessment of test-match correspondence. For each performance domain, the single field-test variable showing the highest Pearson correlation with the corresponding match outcome was identified to examine domain-specific test performance.

For between-group comparisons (Non-CI vs. CI), Welch's t-tests were used because they do not assume equal variances and are recommended for heterogeneous groups in applied sports science (12). Effect sizes were expressed as Hedges’ g, which corrects for small sample bias and facilitates interpretation of practical significance (13). To account for multiple comparisons across test parts within each domain, both Holm-Bonferroni (family-wise error control) and Benjamini-Hochberg false discovery rate (FDR) corrections were applied. FDR was used as the primary criterion, as it balances Type-I error control with sensitivity to detect meaningful group differences, while Holm is reported as a more conservative benchmark. Reporting both approaches allows sensitivity (FDR) and conservativeness (Holm) to be transparently balanced.

This analytical strategy enables robust evaluation of both the strength of association and the quality of agreement between field tests and match outcomes and provides a transparent framework for examining subgroup differences between athletes with and without coordination impairment. The sample size, while relatively large for elite wheelchair rugby research, remains modest, particularly for subgroup analyses. This may affect the stability of estimated effect sizes.

## 3 Results

**Figure 1:**
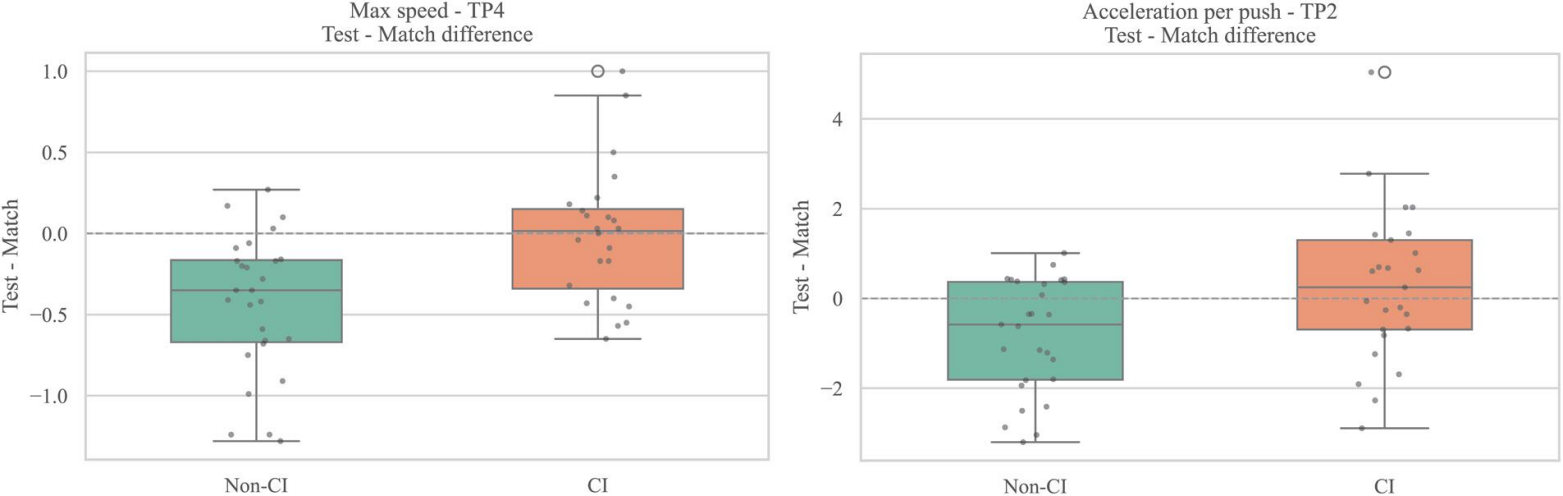
Bland–Altman plot of maximal wheelchair speed (testMax) comparing field-based test outcomes with match-derived performance. The solid line represents the mean bias, and dashed lines indicate the 95% limits of agreement. Data are shown separately for athletes with (CI) and without coordination impairment (Non-CI). Bland–Altman plots for all other test parts are provided in the Supplementary Materials.

All fifty-two athletes were included (Non-CI: *n* = 27; CI: *n* = 25), but one athlete missed the 180° turns in the field tests (TP5-8). Associations between field-test and match outcomes were examined across the five performance domains. Table 2 presents domain-level correlations (r), concordance (CCC), and Bland–Altman agreement statistics using the maximal values across test parts (TestMax). Figure 1 shows the example Bland–Altman for the maximum speed; all other plots are included in the Supplementary Materials.

**Table 2:** Association and agreement between match-derived outcomes and domain-level TestMax values. TestMax was computed as the row-wise maximum across all corresponding field-test parts (TPs) within each performance domain. Reported are sample size (*n*), Pearson's correlation coefficient (*r*) with lower and upper 95% confidence interval bounds (*r* lo, *r* hi), Lin's concordance correlation coefficient (CCC) with 95% confidence interval, mean bias (Test−Match), and the lower and upper 95% limits of agreement (LoA).

Domain	*n*	*r*	*r* lo	*r* hi	CCC	Bias	LoA low	LoA high
Acceleration per push [m/s^2^]	52	0.53	0.30	0.70	0.53	0.09	−3.01	3.18
Max speed [m/s]	52	0.88	0.80	0.93	0.71	0.55	−0.22	1.32
Max rotational speed [°/s]	51	0.80	0.67	0.88	0.55	−65	−153	22
Mean acceleration [m/s^2^]	52	0.68	0.50	0.80	0.04	3.36	0.41	6.31
Mean rotational acc. [°/s^2^]	52	0.56	0.34	0.72	0.02	498	122	875

### 3.1 Domain level correspondence

Maximal forward speed showed the strongest association between field-test-derived maximal capacity (TestMax) and match-derived outcomes (*r* = 0.88), with moderate concordance (CCC = 0.71) and a small positive bias (+0.55 m/s). Maximal rotational speed also showed strong associations with match rotational speeds (*r* = 0.80), with moderate concordance (CCC = 0.55) and a negative bias (−65 deg/). The limits of agreement ranged from −153 to 22 deg/s. Mean forward acceleration (*r* = 0.68) and mean rotational acceleration (r = 0.56) demonstrated moderate associations but near zero CCC values and broad LoA, suggesting limited absolute agreement.

For both mean acceleration domains, Bland–Altman plots revealed proportional bias, with Test-Match differences increasing at higher average accelerations (Supplementary Figure 1). Field tests therefore increasingly overestimated match accelerations at higher intensities.

### 3.2 Best test items per domain

Table 3 summarizes the highest-correlating test part (TP) for each domain. For maximal forward speed, the 20 m sprint (TP1) showed excellent correspondence with match values (*r* = 0.878), a small positive bias (+0.533 m/s), and relatively narrow LoA (−0.230–1.296 m/s). Maximal rotational speed was best captured by the 180° on-the-spot left turn (TP6), yielding *r* = 0.796 and a large negative bias (−79 deg/s).

**Table 3:** Association and agreement between match-derived outcomes and the best-performing field-test item (TP-specific) per performance domain. For each domain, the single field-test variable showing the highest Pearson correlation (*r*) with the corresponding match outcome was selected. Reported are sample size (*n*), Pearson's r with lower and upper 95% confidence interval bounds (*r* lo, *r* hi), Lin's concordance correlation coefficient (CCC) with 95% confidence interval, mean bias (Test−Match), lower and upper 95% limits of agreement (LoA), and the corresponding test part (TP).

Domain	*n*	*r*	*r* lo	*r* hi	ccc	Bias	LoA low	LoA high	TP
Acceleration per push [m/s^2^]	51	0.54	0.31	0.71	0.43	−0.87	−3.53	1.78	3 Interval
Max forward speed [m/s]	52	0.88	0.80	0.93	0.71	0.53	−0.23	1.30	1 20 m sprint
Max rotational speed [°/s]	51	0.80	0.67	0.88	0.49	−79	−169	10	6 180° L
Mean acceleration [m/s^2^]	52	0.72	0.55	0.83	0.05	2.67	−0.02	5.35	1 20 m sprint
Mean rotational acc. [°/s^2^]	52	0.62	0.42	0.77	0.15	93	19	167	9 X-test no ball

Mean forward acceleration was again most strongly reflected by the 20 m sprint (TP1, *r* = 0.716), whereas mean rotational acceleration was best approximated by the X-test without ball (TP9, *r* = 0.624). Acceleration per push showed only modest correspondence, with the interval sprint (TP3) providing the highest correlation (*r* = 0.539) and wide, heterogeneous LoA (−3.53–1.78 m/s^2^).

### 3.3 Group differences in test-match discrepancies

**Figure 2:**
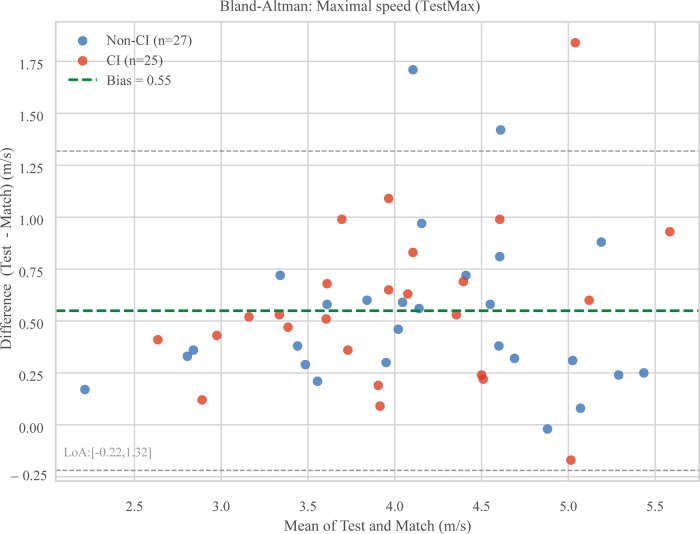
Test-match discrepancies for representative test parts from the domains of maximal wheelchair speed (TP4) and acceleration per push (TP2). Boxplots illustrate the distribution of per-athlete discrepancies for athletes with coordination impairment (CI) and without coordination impairment (Non-CI), with individual data points overlaid. Positive values indicate higher test performance relative to match performance. Additional results are provided in the Supplementary Materials.

Per-athlete Test-Match discrepancy scores were compared between Non-CI and CI athletes across all tests of the field-test battery (TPs; Table 4 and Supplementary Table 1 and Supplementary Figure 2). Representative differences in domains showing significant between-group effects are illustrated in Figure 2.

**Table 4:** Summary of significant condition × group effects (Non-CI vs. CI) on the Test-Match discrepancy across prespecified test parts (TPs). Displayed are Welch's *p*, Benjamini-Hochberg FDR (*q*, within domain), Holm-adjusted *p* (within domain), Hedges’ *g*, and the direction indicating which group showed a larger absolute discrepancy. Full per-TP statistics for all domains are provided in Supplementary Table 1*.*

Domain	TP	Welch *p*	*q* bh	*p* holm	Hedges *g*	Direction
Max speed	4 Slalom	0.001	0.005	0.005	−0.984	abs(Non-CI) > abs(CI)
Max speed	2 12 m sprint	0.005	0.013	0.027	−0.799	abs(Non-CI) > abs(CI)
Max speed	3 Interval	0.007	0.013	0.027	−0.767	abs(Non-CI) > abs(CI)
Max speed	9 X-test no ball	0.018	0.027	0.053	−0.675	abs(Non-CI) > abs(CI)
Max acc. per push	2 12 m sprint	0.013	0.034	0.078	−0.716	abs(Non-CI) > abs(CI)
Max acc. per push	1 20 m sprint	0.017	0.034	0.086	−0.686	abs(Non-CI) > abs(CI)
Max acc. per push	4 Slalom	0.017	0.034	0.086	−0.683	abs(Non-CI) > abs(CI)

For maximal forward speed, significant between-group differences after false discovery rate (FDR) correction were observed in several test parts. The largest effects were found for the combined sprint, turning, and slalom-like movement task (TP4, *q* = 0.0049). Significant differences were also present for the 12 m sprint with a complete stop (TP2, *q* = 0.013), for the 12 m sprint with repeated stop-go transitions (TP3, *q* = 0.013), and for the X-test including ball handling (TP10, *q* = 0.027).

For acceleration per push, significant group differences after FDR correction were identified in the 20 m straight-line sprint (TP1), the 12 m sprint (TP2), and the slalom (TP4, all *q* ≈ 0.034). However, none of these effects remained significant following the more conservative Holm correction. In contrast, no significant between-group differences were observed for mean forward acceleration, mean rotational acceleration, or maximal rotational speed in any test component, regardless of whether the task emphasized straight-line sprinting, stop-start behavior, or rotational movements.

Across all test components showing significant effects, the absolute Test-Match discrepancy was consistently larger in the Non-CI group (see Figure 2 and Supplementary Figure 1). This might indicate that performance outcomes obtained during field-based tests corresponded more closely with match-derived performance measures in CI athletes than in Non-CI athletes.

### 3.4 Trunk sensor outcomes

Trunk mounted IMU data revealed marked group differences during early sprint tasks (Table 5). Non-CI athletes showed larger mean trunk angles (TP1: *g* = 0.53) and substantially greater backward and forward trunk accelerations (TP1 backward: *g* = 1.20; TP2 backward: *g* = 0.94; TP1 forward: *g* = 0.87). The standard deviations in backward acceleration (extension) are larger in the CI group, whereas they are smaller in mean trunk angle. These results demonstrate pronounced active trunk range of motion differences between groups, consistent with prior findings linking trunk impairment to mobility performance in WR.

**Table 5:** Between-group differences in trunk-sensor outcomes during field tests. Mean trunk angle (flexion/extension; positive toward extension), forward trunk acceleration relative to the wheelchair, and backward trunk acceleration relative to the wheelchair are summarized for Non-CI and CI athletes during TP1 and TP2 (early sprint tasks). Values are mean ± SD per group, with Hedges’ *g* (bias-corrected standardized mean difference) and Welch's t-test *p*-values reported for group contrasts.

Variable	TP	Non-CI			CI				
	Sprint	*n*	mean	sd	*n*	mean	sd	Hedges *g*	Welch *p*
Backward acc per push [m/s^2^]	1 20m	27	−1.19	0.64	24	−2.18	0.96	1.1992	0.0001
Backward acc per push [m/s^2^]	2 12m	27	−1.21	0.62	25	−2.05	1.09	0.9422	0.0017
Forward acc per push [m/s^2^]	1 20m	27	1.06	0.54	24	1.58	0.64	−0.8744	0.0030
Forward acc per push [m/s^2^]	2 12m	27	1.17	0.75	25	1.36	0.60	−0.2690	0.3258
Mean trunk angle [^o^]	1 20m	27	52.4	21.7	24	42.2	15.3	0.5289	0.0567
Mean trunk angle [^o^]	2 12m	26	52.9	21.1	25	47.5	15.6	0.2846	0.3048

### 3.5 Supplementary analyses: role of classification level

Supplementary analyses (see Supplementary Table 2) were performed to assess whether classification level confounded or moderated the primary associations between field-test and match-derived performance outcomes. These analyses were intended to evaluate the robustness of impairment-group comparisons rather than to examine classification as a primary explanatory factor. Classification showed expected positive associations with both match and field-test outcomes, but interaction effects with CI status were non-significant across all models, indicating that classification did not moderate or confound any observed group differences.

## 4 Discussion

Taken together, these results highlight systematic differences in how field-test outcomes translate to match-derived performance across mobility domains and impairment types. This study evaluated the ecological validity and test–match correspondence of a comprehensive set of wheelchair rugby field tests against match-derived mobility metrics and examined whether relationships differed between CI and Non-CI athletes. The results provide three central insights. First, domain-specific field tests show strong associations with match mobility performance. Second, test-match agreement differs fundamentally between metrics reflecting maximal performance and context-sensitive domains (e.g., acceleration metrics). Third, this agreement is systematically impairment-dependent, with closer correspondence between test and match outcomes in athletes with CI.

It is important to note that field-based test outcomes and match-derived performance metrics reflect partially distinct constructs. Field tests primarily capture maximal physical capacity under standardized conditions, whereas match-derived measures reflect context-dependent performance shaped by tactical, environmental, and interactional constraints.

### 4.1 Domain-specific validity of field tests for match mobility

The 20 m sprint emerged as the most valid predictor of match maximal forward speed, aligning with prior work highlighting linear sprint performance as a central determinant of WR mobility. Its small positive bias and relatively narrow limits of agreement indicate that this test offers both strong linear association and reasonable absolute agreement. Rotational capacity was best identified by an isolated 180° on-the-spot turn (TP6), which appears to reflect the specific rotational demands required during WR competition. At the domain level, maximal rotational speed showed a strong association between test- and match-based outcomes (*r* ≈ 0.80), accompanied by a moderate concordance coefficient and a systematic negative bias, indicating lower values in the standardized tests. This pattern can be explained by the dependence of rotational velocity on linear velocity at the onset of a turn. Previous work has shown that, under wheelchair rugby equipment and testing constraints, the velocity of an 180° turn is primarily determined by the approach speed into the turn (14). In match play, turns are typically initiated at higher linear velocities than in standardized “turn-on-the-spot” tests such as TP6, leading to higher rotational speeds during games and thus to the observed negative test-match bias despite a strong relative agreement.

In contrast, acceleration-based domains showed moderate correlations but weak absolute agreement. Both mean forward and rotational acceleration displayed proportional bias, with test-match differences increasing as average accelerations became larger. This finding reveals that field tests systematically elicit higher accelerations than those achieved in match play at the upper performance range. Accelerative output has a strong relationship with trunk impairment (14–16). Contextual constraints in match settings, such as defensive pressure, contact, and tactical pacing, that may limit the use of trunk function and may consequently constrain the extent to which maximal accelerative capacity can be expressed during gameplay. As such, acceleration metrics should be interpreted primarily as indicators of physical capacity rather than direct proxies of in game acceleration behavior.

Similarly, acceleration per push showed only modest associations with match equivalents, likely due to the complex interaction between propulsion technique, trunk stability, and opponent interference during matches. Consequently, this metric may be most suitable for evaluating propulsion strategies or impairments rather than for predicting match demands. Rather than diminishing their relevance, this domain-specific limitation clarifies the distinct role of acceleration metrics as indicators of maximal capacity and impairment expression, rather than as proxies for match-equivalent output.

### 4.2 Impairment-related differences in test-match agreement

CI athletes consistently demonstrated smaller absolute discrepancies between test- and match-derived outcomes across the test parts showing significant effects. In contrast, Non-CI athletes exhibited larger deviations, particularly for maximal speed and acceleration per push. These findings may indicate that standardized field tests provide a closer approximation of typical in-game mobility performance in athletes with CI, whereas in Non-CI athletes test outcomes tend to underestimate the intensities reached during match play or reflect more variable performance behavior. Importantly, the observed group differences should not be attributed solely to CI status, as the Non-CI group comprises a heterogeneous set of impairments with potentially different functional and biomechanical consequences.

These group differences suggest that the ecological meaning of standardized field-based tests is not uniform across impairment types. For CI athletes, performance expressed during isolated or standardized tasks appears to generalize well to the dynamic and interactive game environment. For Non-CI athletes, stronger postural control and movement adaptability may enable the expression of higher or more situation-specific intensities during match play than can typically be elicited under standardized testing conditions. The present data does not allow these underlying mechanisms to be fully disentangled. However, the consistent direction of the discrepancy—lower test values relative to match-derived values—suggests that systematic underestimation of match intensity by standardized tests is a plausible contributor, particularly in athletes with greater movement adaptability.

Importantly, these findings do not challenge the use of field-based tests *per se* but rather indicate that the interpretation of their established validity depends on impairment characteristics. This distinction becomes particularly relevant when considering how performance observations are interpreted within the wheelchair rugby classification framework. Although the classification procedure is identical for all athletes, combining medical assessment, observation of standardized activities, and on-court observation, the relative informational contribution of these components may differ depending on impairment characteristics. In impairments for which objective, impairment-specific medical tests are available (e.g., muscle strength, range of motion), observed activity performance primarily contextualizes or confirms the medical findings. In contrast, for coordination impairment, where objective quantification of impairment severity in classification is currently limited, greater emphasis is placed on functional performance observed during standardized activities and game play.

This interpretation is further supported by the observed group differences in trunk control. Trunk sensor data showed larger forward and backward trunk accelerations and greater trunk angles in Non-CI athletes, with less variability than in CI athletes, reflecting superior postural control and a greater capacity to contribute trunk motion to propulsion. These trunk-related capacities may enable Non-CI athletes to express higher intensities during match play than are elicited in standardized test settings, contributing to the larger discrepancies between test- and match-derived performance measures observed in this group. Importantly, trunk sensor outcomes were not modeled as direct predictors of Test-Match discrepancy and therefore support an explanatory interpretation at group level rather than a causal inference at the individual level. These interpretations should be considered exploratory, as trunk-related measures were not directly modeled as predictors of test–match discrepancies, and causal mechanisms cannot be inferred from the present design.

Although classification level was, as expected, related to many individual performance measures, it did not explain or moderate any of the observed impairment-related differences in test-match agreement. This may indicate that the CI versus Non-CI distinctions reported in this study reflect impairment-specific performance characteristics rather than effects of classification level or classification-related confounding. In this sense, the classification analyses serve primarily to support the robustness of the main findings by ruling out classification as an alternative explanation for the observed Test-Match agreement patterns. Importantly, these findings do not indicate differences in quality, rigor, or application of the classification system across impairment types. Rather, they highlight that standardized performance observations may reflect impairment-related performance characteristics differently, even within a uniform classification framework. From a longer-term perspective, the strong discriminatory value of trunk sensor metrics observed in this study suggests potential avenues for future research aimed at strengthening evidence-based classification, without implying immediate changes to current classification procedures.

### 4.3 Practical implications

The results support prioritizing the 20 m sprint for assessing maximal forward speed and isolated 180° turns for evaluating rotational capacity. Conversely, acceleration metrics may be best used to assess maximal capacity, trunk usage, or propulsion technique rather than approximate match level accelerations. For applied practitioners, trunk sensor data offers valuable insight into movement strategy and impairment related limitations and may strengthen athlete profiling and evidence-based classification.

The ecological meaning of field-based tests differs between impairment types. In athletes with CI, test outcomes showed relatively small Test-Match discrepancies and can therefore be interpreted as reasonably representative of in-game mobility performance. In contrast, for non-CI athletes, field tests primarily reflect maximal capacity and tend to underestimate the intensities achieved during match play.

### 4.4 Limitations

Several limitations should be considered when interpreting the present findings. Although the sample represents elite wheelchair rugby athletes, the overall size remains modest, which may affect statistical power and the stability of effect size estimates. In addition, the Non-CI group comprised a heterogeneous range of impairments with differing functional consequences, and residual confounding related to impairment type and classification characteristics cannot be excluded. Furthermore, both field-test and match-derived outcomes were obtained using the same IMU technology and analytical pipeline. While this enabled direct comparability, shared-method variance may have contributed to the observed associations. It should also be recognized that match-derived metrics reflect context-dependent performance shaped by tactical, environmental, and interactional factors, rather than an independent gold standard. The use of TestMax provides an estimate of maximal expressed capacity but does not capture within-athlete variability or performance consistency. Finally, the cross-sectional design and absence of multivariable modelling limit the ability to disentangle underlying mechanisms or draw causal inferences, particularly regarding impairment-related differences and trunk-related findings.

## 5 Conclusion

This study demonstrates that simple, well defined WR field tests validly capture key aspects of maximal mobility capacity that translate, to varying extents, to match mobility performance, with the 20 m sprint and isolated 180° turn serving as the strongest predictors of forward and rotational match speed. However, acceleration-based outcomes show proportional bias and limited absolute agreement, indicating that they reflect maximal physical capacity rather than in game activities. CI athletes exhibited smaller Test-Match discrepancies than Non-CI athletes, suggesting that field-test outcomes are more representative of match performance for athletes with CI. Trunk sensor measures revealed substantial group differences and may provide valuable additional information for performance assessment and classification. Together, these findings highlight the importance of domain specific interpretation of field tests and support the integration of trunk related measures into future evidence-based classification and athlete evaluation frameworks.

## Data Availability

The datasets generated and/or analyzed during the current study are not publicly available because of the high risk of participant re-identification, given the small and specific sample of individual athletes. To protect participant privacy and comply with ethical and legal requirements, the data cannot be shared. Requests to access the datasets should be directed to: r.m.a.vanderslikke-1@tudelft.nl.
