# Supplementary material for: Linking field-based mobility tests to match-derived performance in wheelchair rugby: understanding test-match agreement across impairment types
Source: Front Sports Act Living. 2026 Jul 1;8:1878412. doi: 10.3389/fspor.2026.1878412 (PMC13371710; doi:10.3389/fspor.2026.1878412)
Supplement: Supplementary file 1 [file Datasheet1.docx]

Supplementary Material

# Supplementary Data

Figure Sx Bland-Altman plots

*
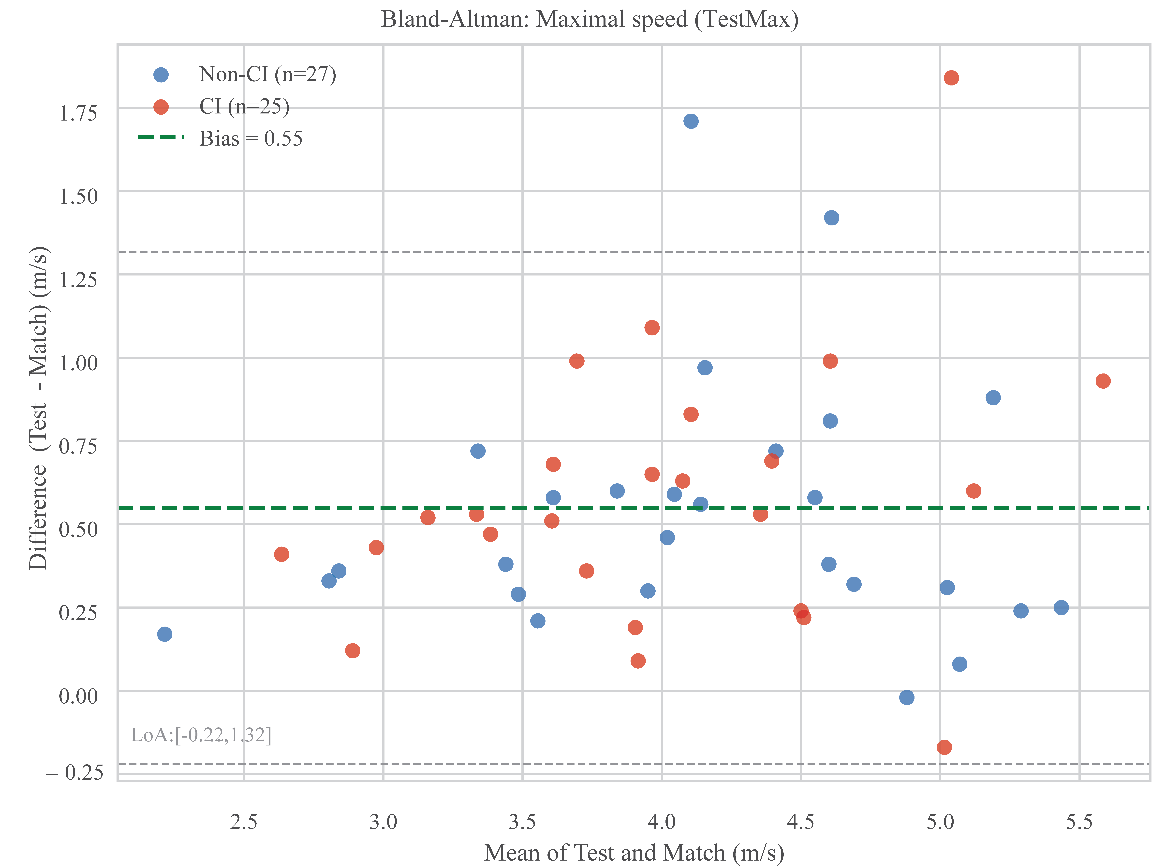
*

*
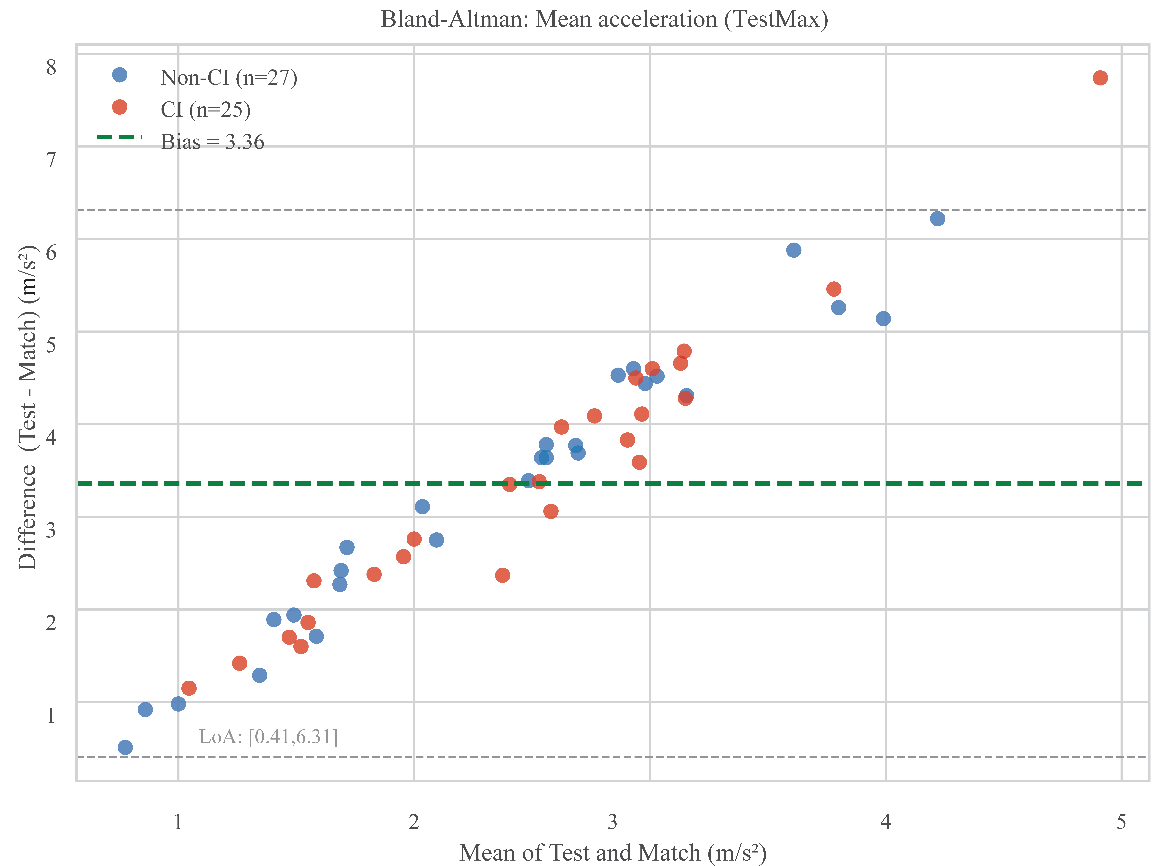
*

*
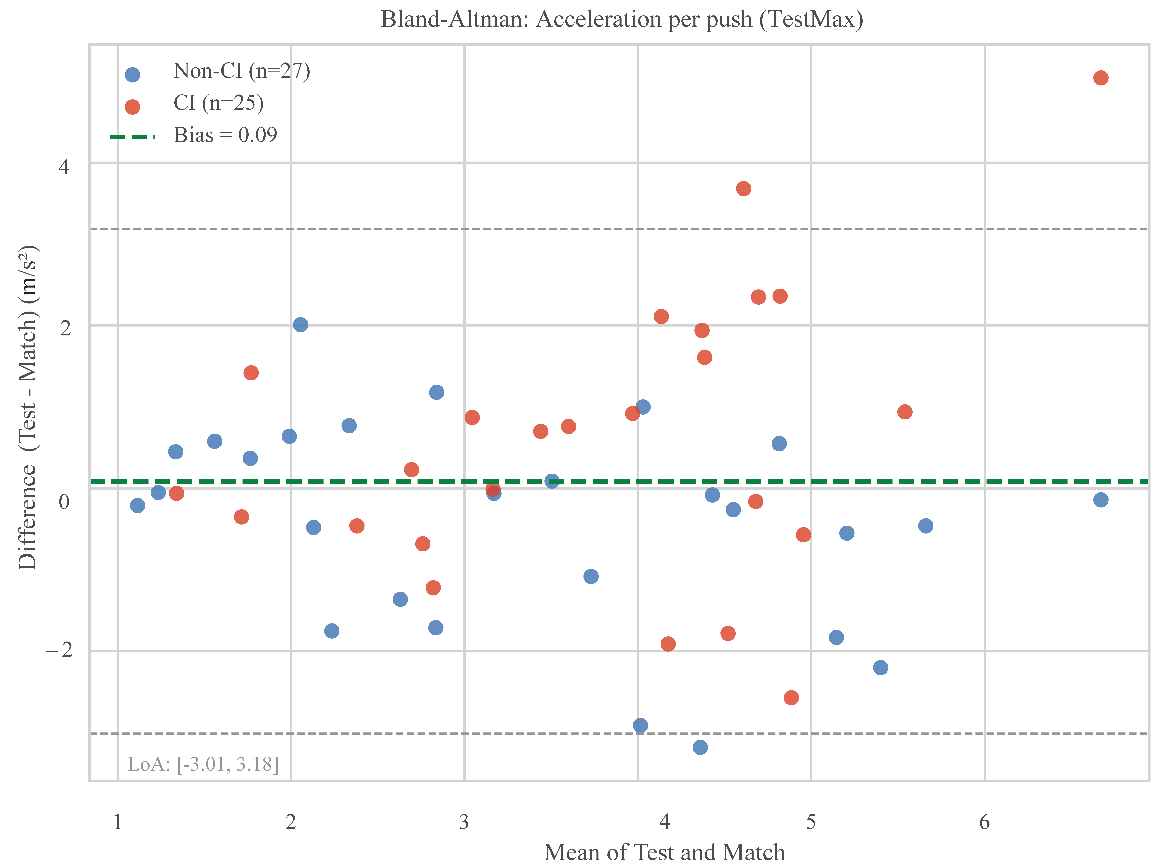
*

*
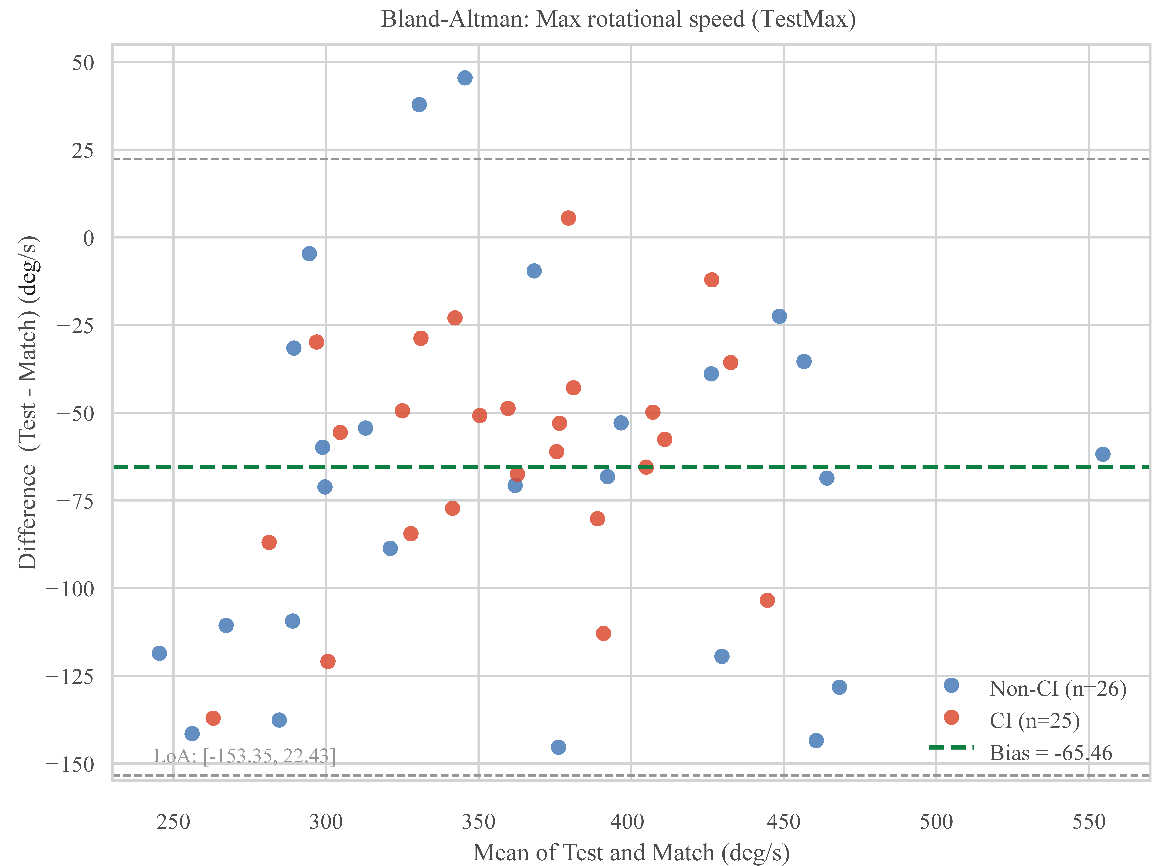

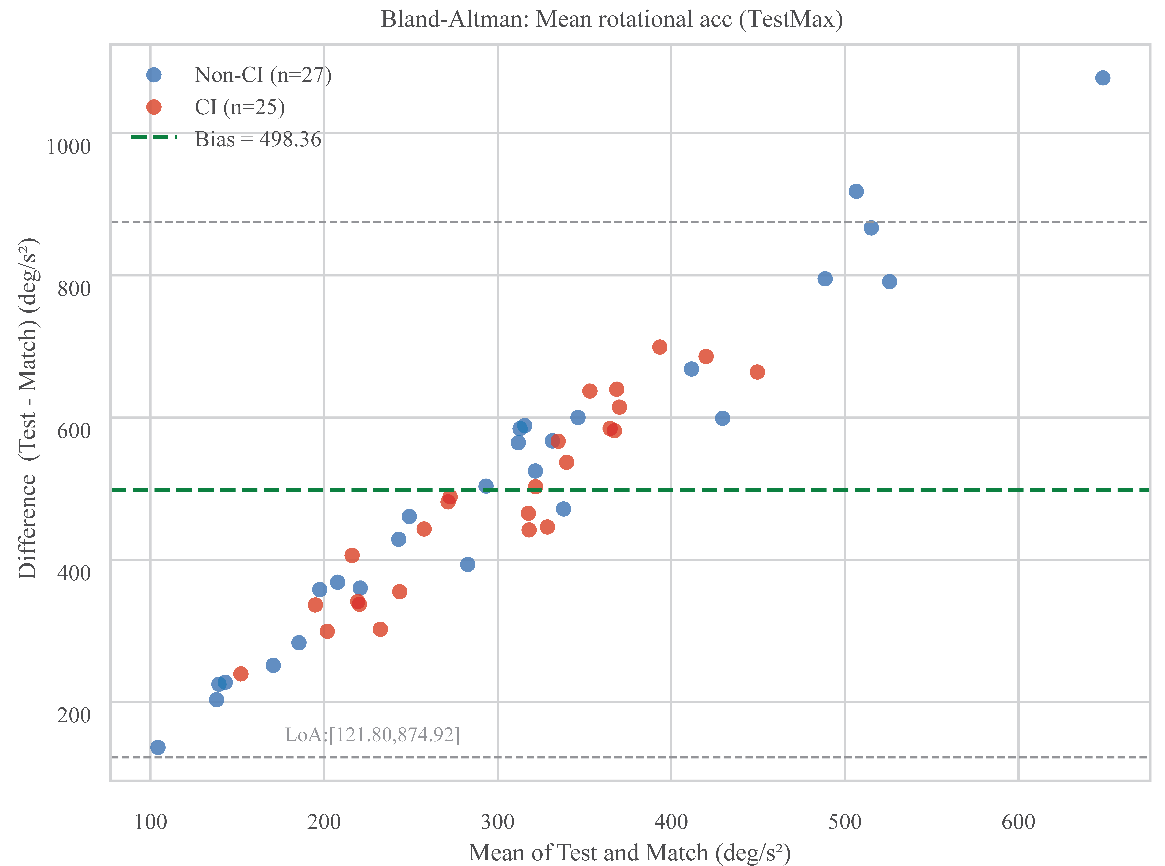
*

Table Sx. Group differences in the Test–Match discrepancy across prespecified test parts (TPs) by domain. For each domain, athlete‑wise difference scores (Test − Match) were compared between Non‑CI and CI using Welch’s t-tests. Shown are n per group, group means ± SD, Hedges’ g, Welch’s p, and multiplicity‑adjusted values (BH‑FDR q and Holm p) computed within domain across the prespecified TPs. Positive values indicate higher Test than Match.

|  |  |  | Non-CI | | | CI | | |  |  |  |  |
| --- | --- | --- | --- | --- | --- | --- | --- | --- | --- | --- | --- | --- |
| Domain | **tp** | **unit** | **n** | **mean** | **sd** | **n** | **mean** | **sd** | **hedges g** | **welch p** | **p holm** | **q bh** |
| Acc per push | 1 | m/s^2^ | 27 | -0.51 | 1.27 | 25 | 0.55 | 1.77 | -0.69 | 0.02 | 0.09 | 0.03 |
| Acc per push | 2 | m/s^2^ | 27 | -0.82 | 1.27 | 25 | 0.28 | 1.73 | -0.72 | 0.01 | 0.08 | 0.03 |
| Acc per push | 3 | m/s^2^ | 26 | -1.09 | 1.47 | 25 | -0.64 | 1.20 | -0.33 | 0.24 | 0.48 | 0.29 |
| Acc per push | 4 | m/s^2^ | 27 | -0.81 | 1.29 | 25 | 0.20 | 1.63 | -0.68 | 0.02 | 0.09 | 0.03 |
| Acc per push | 9 | m/s^2^ | 27 | -1.01 | 1.32 | 25 | -0.16 | 1.49 | -0.59 | 0.04 | 0.11 | 0.06 |
| Acc per push | 10 | m/s^2^ | 26 | -1.55 | 1.47 | 24 | -1.53 | 1.31 | -0.02 | 0.96 | 0.96 | 0.96 |
| Max rot. speed | 3 | deg/s | 24 | -249 | 56 | 24 | -238 | 45 | -0.21 | 0.47 | 1.00 | 0.89 |
| Max rot. speed | 4 | deg/s | 24 | -171 | 51 | 24 | -167 | 35 | -0.09 | 0.77 | 1.00 | 0.89 |
| Max rot. speed | 5 | deg/s | 23 | -1 | 56 | 24 | 20 | 41 | -0.42 | 0.16 | 1.00 | 0.89 |
| Max rot. speed | 6 | deg/s | 23 | 11 | 58 | 24 | 4 | 36 | 0.16 | 0.59 | 1.00 | 0.89 |
| Max rot. speed | 7 | deg/s | 23 | -53 | 47 | 24 | -52 | 27 | -0.04 | 0.90 | 1.00 | 0.90 |
| Max rot. speed | 8 | deg/s | 23 | -51 | 48 | 24 | -62 | 32 | 0.25 | 0.40 | 1.00 | 0.89 |
| Max rot. speed | 9 | deg/s | 24 | -128 | 52 | 24 | -132 | 42 | 0.08 | 0.77 | 1.00 | 0.89 |
| Max rot. speed | 10 | deg/s | 24 | -125 | 54 | 24 | -141 | 35 | 0.34 | 0.23 | 1.00 | 0.89 |
| Max speed | 1 | m/s | 27 | 0.18 | 0.40 | 24 | 0.32 | 0.41 | -0.35 | 0.21 | 0.42 | 0.25 |
| Max speed | 2 | m/s | 27 | -0.52 | 0.47 | 24 | -0.16 | 0.41 | -0.80 | 0.01 | 0.03 | 0.01 |
| Max speed | 3 | m/s | 27 | -1.31 | 0.49 | 24 | -0.97 | 0.33 | -0.77 | 0.01 | 0.03 | 0.01 |
| Max speed | 4 | m/s | 27 | -0.43 | 0.43 | 24 | -0.01 | 0.42 | -0.99 | 0.00 | 0.00 | 0.00 |
| Max speed | 9 | m/s | 27 | -0.89 | 0.53 | 24 | -0.54 | 0.50 | -0.68 | 0.02 | 0.05 | 0.03 |
| Max speed | 10 | m/s | 27 | -1.54 | 0.49 | 24 | -1.46 | 0.46 | -0.17 | 0.55 | 0.55 | 0.55 |
| Mean acc | 1 | m/s^2^ | 27 | 2.47 | 1.25 | 25 | 2.88 | 1.49 | -0.29 | 0.29 | 1.00 | 0.87 |
| Mean acc | 2 | m/s^2^ | 27 | 3.08 | 1.39 | 25 | 3.08 | 1.34 | 0.00 | 0.99 | 1.00 | 0.99 |
| Mean acc | 3 | m/s^2^ | 27 | 3.06 | 1.56 | 25 | 3.12 | 1.41 | -0.04 | 0.88 | 1.00 | 0.99 |
| Mean acc | 4 | m/s^2^ | 27 | 1.84 | 0.81 | 25 | 2.05 | 1.18 | -0.20 | 0.48 | 1.00 | 0.95 |
| Mean acc | 9 | m/s^2^ | 27 | 1.63 | 0.83 | 25 | 1.69 | 0.91 | -0.06 | 0.82 | 1.00 | 0.99 |
| Mean acc | 10 | m/s^2^ | 27 | 0.94 | 0.55 | 25 | 0.62 | 0.51 | 0.59 | 0.03 | 0.20 | 0.20 |
| Mean rot. acc | 3 | deg/s^2^ | 27 | 51 | 42 | 25 | 65 | 35 | -0.37 | 0.18 | 0.73 | 0.47 |
| Mean rot. acc | 4 | deg/s^2^ | 27 | 132 | 58 | 25 | 152 | 61 | -0.33 | 0.24 | 0.73 | 0.47 |
| Mean rot. acc | 9 | deg/s^2^ | 27 | 93 | 39 | 25 | 93 | 37 | 0.01 | 0.98 | 1.00 | 0.98 |
| Mean rot. acc | 10 | deg/s^2^ | 27 | 93 | 40 | 25 | 88 | 45 | 0.12 | 0.67 | 1.00 | 0.89 |

Figure Sx2 Boxplots for Table 4

*
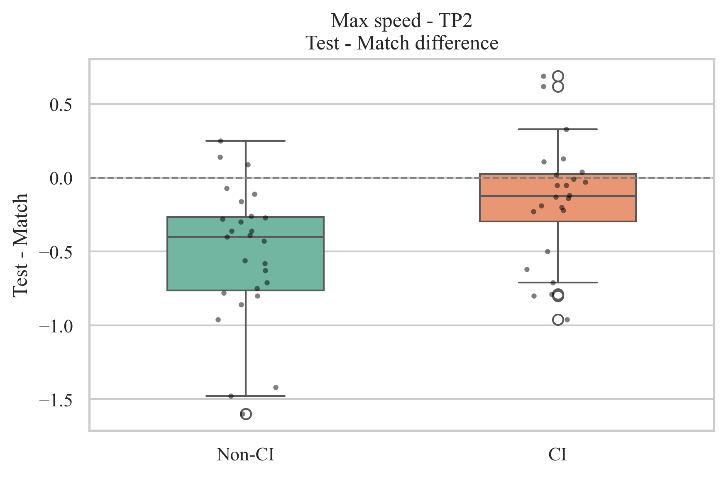

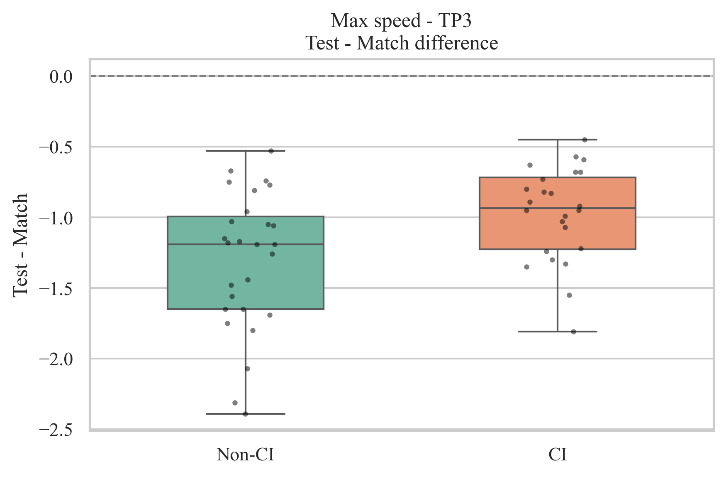
*

*
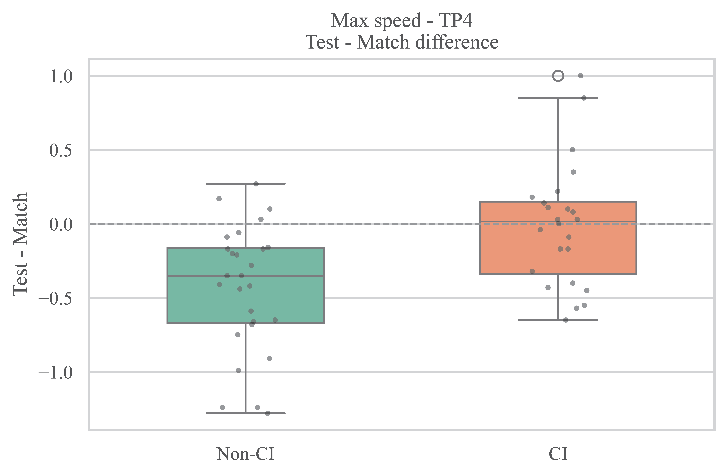

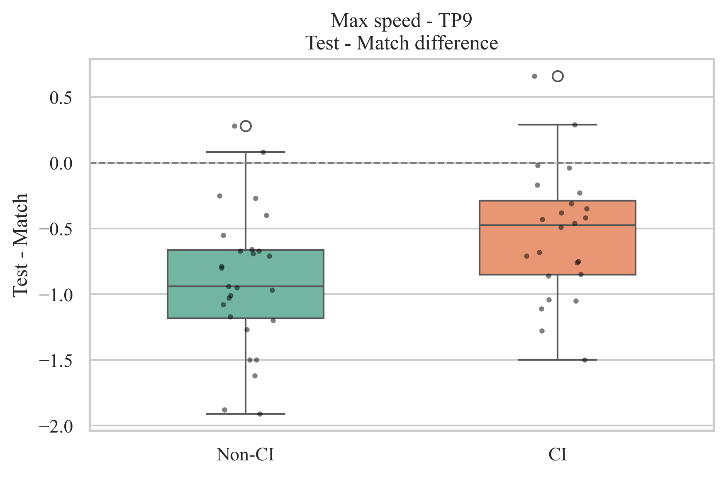

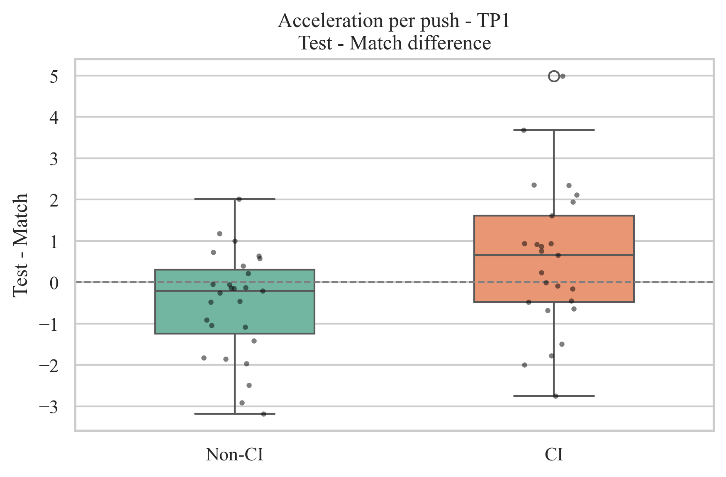

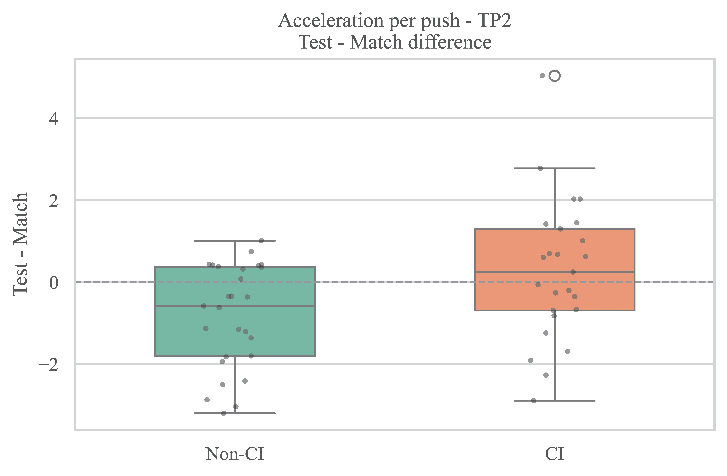

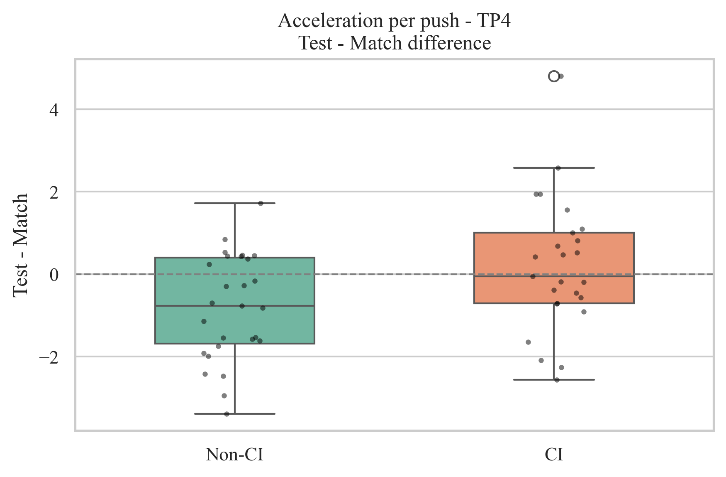
*

**Supplementary Analysis: Association Between Classification Points and Performance Outcomes**

To verify whether functional classification explained additional variance relevant to the comparison between athletes with and without coordination impairment (Non‑CI vs CI), supplementary analyses were performed. Associations between classification points and (1) match‑derived mobility metrics, (2) field test outcomes across all test parts (TPs), and (3) trunk‑sensor variables were examined. Pearson and Spearman correlations were computed, and linear regression models including the interaction term Classification × CI status were fitted for match‑performance variables.

Across all analyses, classification showed the expected positive relationships with performance outcomes, particularly for maximal forward speed and complex field‑test tasks. However, interaction effects with CI status were consistently non‑significant, indicating that the slope of the relationship between classification and performance did not meaningfully differ between Non‑CI and CI athletes. As such, classification did not confound nor moderate the observed group effects in the main study.

Therefore, the classification–performance associations are consistent with previously published work and do not alter the interpretation of the Non‑CI vs CI comparisons presented in the main manuscript.

| Variable | Type | n | Pear-son r | Spear-man r | Beta classifi-cation | Interpretation |
| --- | --- | --- | --- | --- | --- | --- |
| Max speed | Match | 51 | 0.771 | 0.768 | 0.735 | Strong association; no CI-interaction |
| Mean acceleration | Match | 52 | 0.572 | 0.625 | 0.161 | Moderate; similar slopes Non-CI/CI |
| Max rotational speed | Match | 48 | 0.564 | 0.569 | 37.067 | Positive link; no moderation |
| Best TP max speed (TP1) | Field test | 52 | 0.805 | 0.798 |  | 20m sprint reflects class gradient |
| Best TP max rotational speed (TP6) | Field test | 51 | 0.763 | 0.811 |  | Rotation task scales with class |
| Best TP mean acceleration (TP3) | Field test | 52 | 0.766 | 0.751 |  | Stop-go relates to class points |
| Trunk angle (TP1) | Trunk | 51 | -0.495 | -0.544 |  | Higher classes show more flexion |
| Forward trunk acceleration (TP1) | Trunk | 51 | 0.513 | 0.522 |  | Higher classes generate greater drive |
